# Toll-Like Receptor 2-Mediated Suppression of Colorectal Cancer Pathogenesis by Polysaccharide A From *Bacteroides fragilis*

**DOI:** 10.3389/fmicb.2018.01588

**Published:** 2018-07-17

**Authors:** Panida Sittipo, Stefani Lobionda, Kyungchul Choi, Ita Novita Sari, Hyog Young Kwon, Yun Kyung Lee

**Affiliations:** Soonchunhyang Institute of Medi-Bio Science, Soonchunhyang University, Cheonan, South Korea

**Keywords:** polysaccharide A, *Bacteroides fragilis*, colorectal cancer, proliferation, migration

## Abstract

The beneficial role of gut microbiota in intestinal diseases has been highlighted recently. *Bacteroides fragilis* found in the human gastrointestinal tract is a well-studied example of a beneficial bacterium that protects against intestinal inflammation. Polysaccharide A (PSA) from *B. fragilis* induces the production of interleukin (IL)-10 from immune cells via Toll-like receptor 2 (TLR2) signaling in animal colitis models. The direct effect of PSA on human colorectal cancer (CRC) cells has not been studied. Here, we report the effect of PSA from *B. fragilis* on CRC pathogenesis in SW620 and HT29 CRC cells and the molecular signaling underlying these effects. We demonstrated that PSA induced the production of the pro-inflammatory cytokine, IL-8, but not IL-10, in CRC cells. PSA inhibited CRC cell proliferation by controlling the cell cycle and impaired CRC cell migration and invasion by suppressing epithelial mesenchymal transition. Moreover, as in the case of other animal intestinal diseases, the protective role of PSA against CRC pathogenesis was also mediated by TLR2. Our results reveal that PSA from *B. fragilis* plays a protective role against CRC via TLR2 signaling.

## Introduction

Commensal bacteria can regulate the colonic epithelial barrier, which is the first line of defense that protects the body from the gastrointestinal environment. Thus, the commensal bacteria might interact with and modulate the intracellular mechanism of colonic epithelial cells. Moreover, probiotic bacteria such as *Bifidobacterium*, which also reside in the gastrointestinal tract, were shown to reduce tumor volume in rats, indicating a potential role of the gut microbiota in tumor suppression (Singh et al., 1997). Indeed, there are trillions of commensal microorganisms residing in the gastrointestinal tract that play an important role in regulating the immune system of the gut. The dysbiosis of commensal microbiota can promote diseases in the host (Sittipo et al., 2018; Zou et al., 2018). *Firmicutes* and *Bacteroidetes* are major components of the commensal microbiota (Ley et al., 2008). In particular, *Bacteroides fragilis* contains polysaccharide A (PSA), which is responsible for its many beneficial health effects. As a zwitterionic capsular polysaccharide, PSA is known as an immunomodulatory bacterial molecule that shows sufficient experimental immune disease protection in several disease models such as those of inflammatory bowel diseases (IBDs) and central nervous system (CNS) demyelinating disease. PSA modulates the immune system by inducing the production of the potent anti-inflammatory cytokine interleukin (IL)-10 from regulatory T cells (Tregs), thereby limiting pathological inflammation in the gastrointestinal tract and to prevent CNS demyelinating disease (Mazmanian et al., 2008; Ochoa-Reparaz et al., 2010b). PSA requires both innate and adaptive immune responses to exert its immuno-protective effect, which presumably occurs through Toll-like receptor 2 (TLR2) recognition. Specifically, PSA acts through TLR2 on Foxp3+ Tregs to activate immunological tolerance (Round et al., 2011). Moreover, IL-10 production was shown to be stimulated in Tregs by plasmacytoid dendritic cells through a TLR2-dependent mechanism (Dasgupta et al., 2014). In addition, PSA has also been shown to stimulate the TLR2-mediated inflammatory response in antigen-presenting cells, leading to activation of interferon-gamma (IFN-γ)-producing Th1 cells (Wang et al., 2006).

Patients with IBDs have increased risk of developing colorectal cancer (CRC) due to an imbalance of the immune cell populations, which leads to the formation of a tumor-supportive microenvironment in the colon (Danese et al., 2011). CRC is one of the leading causes of cancer-related mortality worldwide, and its incidence has been increasing continuously every year (Siegel et al., 2016). CRC develops and progresses over several years, and is associated with a high rate of invasion and metastasis to other organs such as the lymph nodes and liver (Enquist et al., 2014). One of the key factors involved in tumor mobility is epithelial-mesenchymal transition (EMT) (Nadeau-Vallee et al., 2017), which is a part of the metastatic process. During EMT, the cell–cell adhesion molecules are gradually downregulated in epithelial cells, leading to the loss of cell polarity (Zhu et al., 2013; Bates and Mercurio, 2014), which is accompanied by increased expression of mesenchymal marker proteins, such as *N*-cadherin, fibronectin, vimentin, TWIST1, SNAIL, and SLUG (Lamouille et al., 2014). Given that the EMT can accelerate cancer progression, suppression of the EMT process has emerged as a novel and promising strategy for CRC treatment.

Recently, the American Cancer Society stated that early stage CRC can be treated surgically, whereas chemotherapeutic agents are useful for treating patients at a more advanced stage. However, the effectiveness of chemotherapy in CRC is limited due to various factors such as drug resistance, side effects, and toxicities from the synthetic agents (Rabik and Dolan, 2007). Therefore, finding naturally derived molecules is an important aspect of CRC research to develop novel therapeutic agents for minimizing side effects, toxicities, and dysfunction of immunomodulation associated with conventional CRC treatments (Rabik and Dolan, 2007). The commensal microbiota is an ideal candidate for CRC therapy because it has a symbiotic relationship with the host gastrointestinal tract and can directly interact with intestinal epithelial cells. Recently, it has been found that bacterial derived-molecule, enterotoxin-2, from enterotoxigenic *B. fragilis* has an anti-tumor effect in mouse model of CRC (Lv et al., 2017). Although, PSA, which is derived the beneficial bacterium *B. fragilis* (strain NCTC9343), is known to be sufficient for protection against inflammatory diseases, the effect of PSA from *B. fragilis* on CRC pathogenesis remains unknown.

This study demonstrates the inhibitory function of PSA from *B. fragilis* on CRC pathogenesis using CRC cell lines and patient-derived primary-like CRC cells. PSA induces the production of pro-inflammatory cytokine, IL-8, inhibits CRC cell proliferation by controlling cell cycle-related genes, and suppresses CRC cell migration and invasion via inhibition of epithelial mesenchymal transition process. In addition, the inhibitory function of PSA from *B. fragilis* on CRC pathogenesis is mediated by TLR2. These results provide new insights into the influence of the gut microbiota on CRC pathogenesis, and suggest a potential role of PSA as a new candidate for CRC treatment.

## Materials and Methods

### Cell Culture and Purified PSA From *B. fragilis*

The human colon carcinoma cell lines SW620 (ATCC catalog number: CCL-227) and HT29 (KCLB catalog number: 30038) were used in this study. The cells were grown in RPMI-1640 medium (Corning, NY, United States) supplemented with 10% fetal bovine serum (Corning, NY, United States), 1% minimal essential medium non-essential amino acids (Corning, NY, United States), and 1% penicillin/streptomycin (Corning, NY, United States) at 37°C in an atmosphere of 95% air and 5% CO_2_. The medium was replaced every 2 days. Patient-derived primary-like CRC cells were provided by Professor Steven M Lipkin from the Department of Medicine, Weill Cornell College of Medicine (New York, NY, United States). The cells were grown in Dulbecco’s modified Eagle’s medium (DMEM)/F12 medium (Gibco, United States) supplemented with 1% penicillin/streptomycin (Corning, NY, United States), 2% N2 supplement (Gibco, United States), 0.02% recombinant human epidermal growth factor (HumanZyme, United States), and 0.1% recombinant human fibroblast growth factor basic (Invitrogen, Canada) at 37°C in an atmosphere of 95% air and 5% CO_2_. The cells were dissociated every 7 days. PSA purified from *Bacteroides fragilis* NCTC9343 (Tzianabos et al., 1992) was a gift from Mazmanian laboratory (California Institute of Technology, United States). The purified PSA was dissolved in phosphate-buffered saline (PBS) to obtain a stock solution of 1 mg/ml. PBS was used as a control treatment for this study.

### RNA Extraction and Reverse Transcription-Quantitative Polymerase Chain Reaction (RT-PCR)

The cells (2 × 10^5^) were seeded in a 24-well plate until reaching 60–70% confluence, and then treated with 50 or 100 μg/ml PSA for 6, 12, and 24 h depending on the experiment. RNA was isolated using TRIZOL reagent (Ambion, United States) and converted to cDNA using reverse transcription reagents (TOYOBO, Japan) according to the manufacturer’s protocols. For determination of mRNA expression, RT-PCR was performed using SYBR Green Real time PCR Master Mix Kit (TOYOBO, Japan). The reaction was operated with an ABI StepOne plus real-time PCR machine (Applied Biosystems^TM^, United States) under the following conditions: initial heat activation at 95°C for 1 min, denaturation at 95°C for 30 s, annealing at 60°C for 30 s, and extension at 72°C for 45 s (40 cycles). Primer sequences are listed in **Table 1**.

**Table 1:** List of primer sequences for RT-PCR.

Primers	Sequences (5′ – 3′)
*B2M*_Forward	TGA AGC TGA CAG CAT TCG G
*B2M*_Reverse	CTG CTG GAT GAC GTG AGT AAA
*CXCL8*_Forward	TAC TCC AAA CCT TTC CAC CCC
*CXCL8*_Reverse	CAA CCC TCT GCA CCC AGT TT
*IL1B*_Forward	ACC TGT CCT GCG TGT TGA AA
*IL1B*_Reverse	GGG GAG AAG GTG GTT GTC TG
*IL10*_Forward	CCA CCT CCG CCA ATC TCT CA
*IL10*_Reverse	CTG GGT CTT GGT TCT CAG CTT
*TGFB1*_Forward	TGA CCT GGC CAC CAT TCA
*TGFB1*_Reverse	GTT GGC ATG GTA GCC CTT
*CDH1*_Forward	TTG CAC CGG TCG ACA AAG GAC
*CDH1*_Reverse	TGG ATT CCA GAA ACG GAG GCC
*CDH2*_Forward	GGT GGA GGA GAA GAA GAC CAG
*CDH2*_Reverse	GGC ATC AGG CTC CAC AGT
*SNAI1*_Forward	CTG GGT GCC CTC AAG ATG CA
*SNAI1*_Reverse	CCG GAC ATG GCC TTG TAG CA
*SNAI2*_Forward	TAC CGC TGC TCC ATT CCA CG
*SNAI2*_Reverse	CAT GGG GGT CTG AAA GCT TGG
*CCNB1*_Forward	CGG GAA GTC ACT GGA AAC AT
*CCNB1*_Reverse	AAA CAT GGC AGT GAC ACC AA
*CCND1*_Forward	ACA AAC AGA TCA TCC GCA AAC AC
*CCND1*_Reverse	TGT TGG GGC TCC TCA GGT TC
*CDK2*_Forward	GCT AGC AGA CTT TGG ACT AGC CAG
*CDK2*_Reverse	AGC TCG GTA CCA CAG GGT CA
*CDKN1B*_Forward	TGC AAC CGA CGA TTC TTC TAC TCA A
*CDKN1B*_Reverse	CAA GCA GTG ATG TAT CTG ATA AAC AAG GA

### Enzyme-Linked Immunosorbent Assay (ELISA)

The cells (2 × 10^5^) were plated in a 24-well plate, and treated with 50 or 100 μg/ml PSA upon reaching confluence. After 24 h, the culture supernatants were harvested and the protein levels of IL-8, IL-10, IL-1B, and TGFB1 were assessed by ELISA using the Human IL-8 ELISA MAX^TM^ Deluxe kit (Biolegend, United States), the Human IL-10 ELISA development kit (Mabtech, Sweden), and the human IL-1B and TGFB1 ELISA Ready-SET-Go kit (eBioscience, San Diego, CA, United States) according to the manufacturers’ instructions. The concentration was measured by comparing the optical density value at 450 nm to the standard curve using microplate reader.

### Cell Proliferation Assay

Cell proliferation was evaluated using the MTT assay. The CRC cell lines (5 × 10^3^) were seeded in a 96-well plate, and the monolayer cells were treated with various concentrations of PSA (10, 50, or 100 μg/ml) for 24 and 48 h. For patient-derived primary-like CRC cells, the cell suspension was treated with 50 or 100 μg/ml PSA for 72 h. To neutralize IL-8 in culture media in the presence of PSA, the monoclonal anti-human IL-8 (10 μg/ml, clone 6217, R&D System, United States) was added to the cell culture. At specific time points, the viable cell number was determined using a cell proliferation kit (Roche, Germany) following the manufacturer’s instructions. In brief, 10 μl of MTT labeling reagent was added to the cells for 4 h. The purple formazan crystals were solubilized by adding 100 μl of solubilization solution, and the absorbance of the colored solution was measured on a spectrophotometer at 575 nm (tests) and 650 nm (background) using a microplate reader.

### Western Blotting Analysis

The cells (5 × 10^5^) were seeded into 6-well plate, incubated until reaching 60–70% confluence, and treated with 50 or 100 μg/ml PSA for 24 h. The cell lysates were collected using RIPA Lysis buffer. The protein concentration was quantified by the Bradford protein assay (Bio-Rad Laboratories, Inc., United States). Total protein (30 μg) was separated by 12% sodium dodecyl sulfate-polyacrylamide gel electrophoresis, and electro-transferred onto a 0.45-μm Amersham Hybond polyvinylidene fluoride membrane (GE Healthcare Life Sciences, United Kingdom) using Trans-blot Turbo (Bio-Rad Laboratories, Inc., United States). The non-specific binding was prevented by blocking with 5% skim milk. The membranes were immunoblotted at 4°C overnight with rabbit polyclonal anti-β-actin antibody (AbFrontier, Korea), rabbit monoclonal anti-TLR2 antibody (Abcam, United States), rabbit monoclonal anti-*E*-cadherin antibody (Cell signaling, United States), mouse monoclonal anti-*N*-cadherin antibody (BD, United States), rabbit monoclonal anti-SNAIL antibody (Cell Signaling, United States), or rabbit monoclonal anti-SLUG antibody (Cell Signaling, United States). The membranes were then incubated with the secondary antibody horseradish peroxidase-conjugated anti-rabbit immunoglobulin (Thermo Fisher Scientific, United States) or anti-mouse immunoglobulin (Cell Signaling, United States) for 1 h at room temperature. The specific protein signal was detected by enhanced chemiluminescence using Western ECL substrate (Bio-Rad Laboratories, Inc., United States) and visualized on Amersham Imager 600 (GE Healthcare Life Sciences, United Kingdom). Quantitative analysis of protein expression was measured by the ImageJ software and expressed as the relative optical density.

### Migration and Invasion Assays

A Transwell with an 8.0-μm-pore polycarbonate membrane insert (Corning, NY, United States) without or with coating of 20 μl Matrigel (BD, United States) was used for the migration and invasion assays, respectively. One hundred microliters of serum-free medium containing 2.5 × 10^5^ cells (SW620) or 5 × 10^5^ cells (HT29) was treated with 10, 50, or 100 μg/ml PSA, and then the cells were seeded into each well of the insert. Six hundred microliters of complete medium containing 10% FBS was added outside the Transwell culture insert. After 24 h incubation at 37°C, the insert was gently cleaned using a cotton swab. The cells were fixed with 1% formaldehyde for 15 min, washed twice with PBS, and stained with 0.1% crystal violet for 15 min. The migratory and invasive cells were observed under a microscope (Leica, Germany) and counted by the ImageJ software.

### Short Hairpin RNA (shRNA)-Mediated Knockdown of TLR2

HEK293T cells were transfected with pLKO.1-based lentiviral vectors expressing shRNA targeting TLR2 (TRCN0000057019 or TRCN0000358794) or scrambled control shRNA (Sigma, St. Louis, MO, United States). After 8 h of transfection, the cell supernatants containing lentiviral particles were collected and transduced into the confluent culture of SW620 cells. The virus-containing medium was removed and fresh complete medium was added after 2 days of transduction. The transduced cells were selected by with puromycin (Sigma, United States), and the TLR2-knockdown cells were confirmed by western blot analysis.

### Statistical Analysis

Statistical analysis was conducted using GraphPad software (GraphPad, San Diego, CA, United States) by Student’s *t*-test. Values are presented as mean ± standard deviation. A *p*-value of less than 0.05 was considered statistically significant.

## Results

### PSA Induces the Production of IL-8 by CRC Cells

**FIGURE 1:**
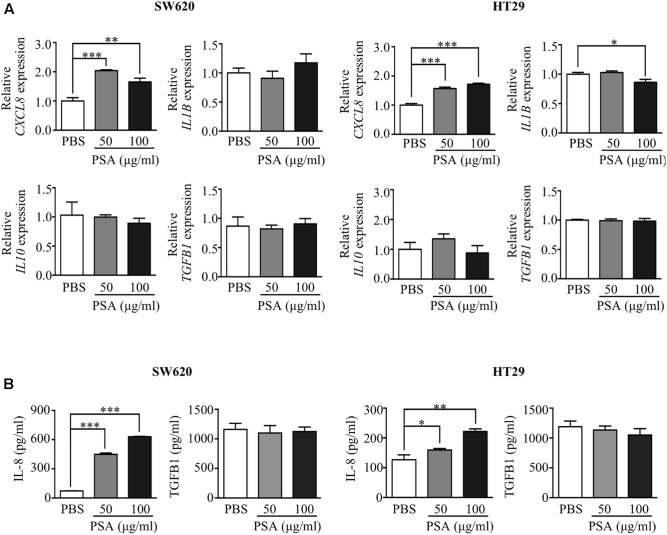
Polysaccharide A (PSA) activates the production of IL-8 by colorectal cancer cells. Human colorectal cancer cell lines SW620 and HT29 were treated with PSA at 50 or 100 μg/ml for 24 h. PBS was used as a control treatment. RNA was isolated from the cells and the relative mRNA expression levels of cytokines (*CXCL8*, *IL1B, IL10*, and *TGFB1*) were quantified by RT-PCR **(A)**. The cell supernatant was harvested to measure the protein concentration of IL-8 (encoded by *CXCL8*) and TGFB1 (encoded by *TGFB1*) by ELISA **(B)**. The data are presented as the mean ± SD from three independent experiments (^∗^*P* < 0.05, ^∗∗^*P* < 0.005, ^∗∗∗^*P* < 0.0005).

*Bacteroides fragilis* PSA is known to induce the production of cytokines from immune cells (Mazmanian et al., 2005, 2008; Ochoa-Reparaz et al., 2010a) and activate the production of IL-8 from embryonic kidney cells (Wang et al., 2006). However, whether PSA stimulates the production of cytokines from CRC cells is still undetermined. To investigate the effect of PSA on the production of cytokines from CRC cells, SW620 and HT29 cells were treated with 50 or 100 μg/ml PSA, and the expression of certain cytokine genes was observed by RT-PCR. The results demonstrated that the CRC cells expressed the cytokines *CXCL8*, *IL1B*, *IL10*, and transforming growth factor beta 1 (*TGFB1*) (**Figure 1A**). The expression of *CXCL8* was significantly upregulated in both CRC cell lines after treatment with 50 and 100 μg/ml PSA for 24 h. However, the expression of *IL1B*, *IL10*, and *TGFB1* was not significantly affected by PSA in SW620 cells. In addition, the expression level of *IL1B* was slightly decreased by 100 μg/ml PSA treatment in HT29 cells. Furthermore, the ELISA results confirmed the dose-dependent upregulation of IL-8, which is encoded by the *CXCL8* gene, while TGFB1 was not significant different in both cell lines after 50 or 100 μg/ml PSA treatment for 24 h (**Figure 1B**). The protein levels of IL-1B and IL-10 were undetectable in both SW620 and HT29 cells. Collectively, these data demonstrated that *B. fragilis* PSA may induce the production of IL-8 by CRC cells.

### PSA Inhibits the Proliferation of CRC Cells via the Suppression of Cell Cycle Progression

**FIGURE 2:**
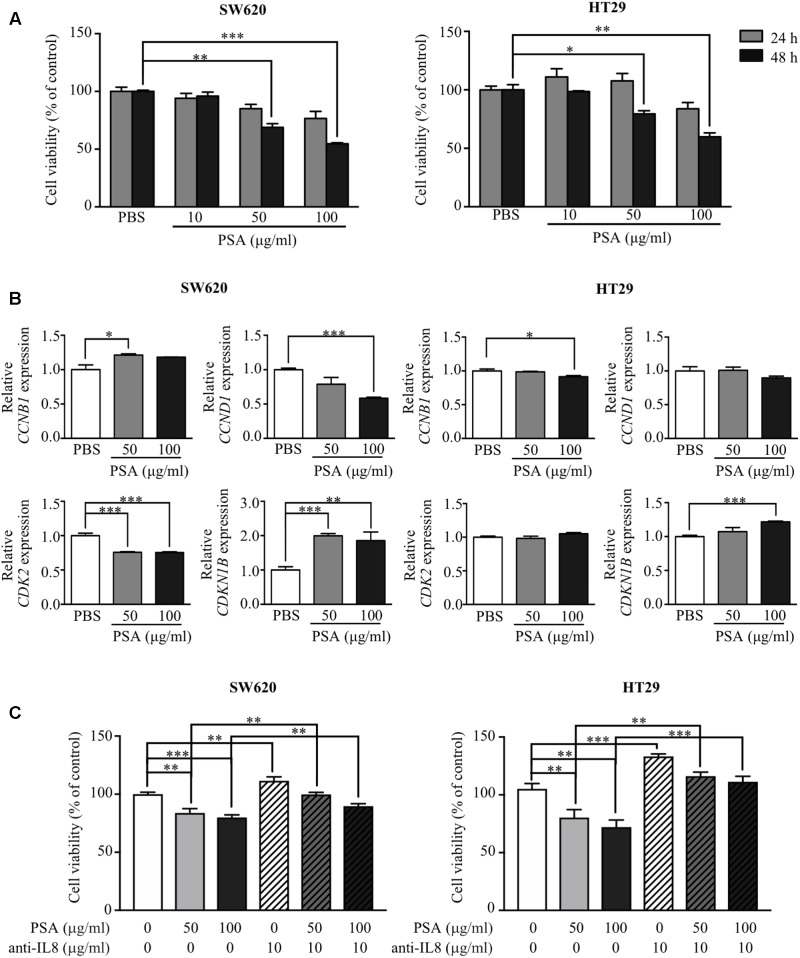
Polysaccharide A inhibits the proliferation of colorectal cancer cells by suppressing the expression of cell cycle-related genes. SW620 and HT29 cells were treated with PSA at 10, 50, or 100 μg/ml, respectively. MTT solution was added to the cell culture to detect cell viability at 24 and 48 h after treatment. The cell viability (percentage) was compared with that of the PBS-treated group (control) **(A)**. SW620 and HT29 cells were treated with PSA at 50 or 100 μg/ml for 24 h; PBS was used as the control treatment. Relative mRNA expression levels of cell cycle-related genes (*CCNB1*, *CCND1*, *CDK2*, and *CDKN1B*) were measured **(B)**. SW620 and HT29 cells were treated with PSA at 50 or 100 μg/ml in the presence or absence of 10 μg/ml anti-human IL-8 neutralizing antibody. After treatment for 48 h, MTT solution was added to the cell culture to detect cell viability **(C)**. The data are shown as the mean ± SD from three independent experiments (^∗^*P* < 0.05, ^∗∗^*P* < 0.005, ^∗∗∗^*P* < 0.0005).

Cancer cells are capable of uncontrolled growth by avoiding checkpoint control mechanism in the cell cycle (Bresciani, 1968; Hanahan and Weinberg, 2011). To investigate the anti-proliferative effect of PSA on CRC cells, a monolayer of SW620 and HT29 cells was treated with various concentration of PSA (10, 50, or 100 μg/ml) for 24 and 48 h, and cell proliferation was detected by the MTT cell proliferation assay at various time points. The results showed that PSA did not significantly affect the viability of either of cell lines after treatment for 24 h. However, PSA treatment for 48 h significantly reduced the number of viable cells in a dose-dependent manner (50 and 100 μg/ml PSA) (**Figure 2A**). The anti-proliferative effect of PSA was also evaluated in patient-derived primary-like CRC cells. The cells were treated with 50 or 100 μg/ml PSA for 72 h, and cell proliferation was determined. Although the growth rate of patient-derived primary-like CRC cells is slower than CRC cell lines, the result showed that patient-derived primary-like CRC cell viability could be significantly reduced by 100 μg/ml PSA treatment (**Supplementary Figure S1**). As cell cycle progression is one of the primary mechanisms related to the control of cell proliferation, we further investigated whether PSA controls cell cycle progression in CRC cells. Monolayer cells were treated with the effective concentrations of PSA (50 and 100 μg/ml PSA) and the expression levels of cell cycle regulatory genes were examined, including the cell cycle-activating genes cyclin B1 (*CCNB1*), cyclin D1 (*CCND1*), and cyclin-dependent kinase 2 (*CDK2*), and the cell cycle-suppressing gene cyclin-dependent kinase inhibitor 1B (*CDKN1B*). As shown in **Figure 2B**, the expression of *CCND1* and *CDK2* was significantly downregulated, while the expression of *CDKN1B* was significantly upregulated in SW620 cells following PSA treatment. In HT29 cells, *CCNB1* expression was slightly downregulated, *CDKN1B* expression was significantly upregulated following 100 μg/ml PSA treatment. These results suggested that *B. fragilis* PSA may suppress the proliferation of CRC cells by inhibition of cell cycle progression.

A previous study showed that IL-8 acts as an autocrine or paracrine signal to suppress non-small cell lung cancer proliferation (Wang et al., 1996). Our data showed that PSA could induce the production of IL-8 by CRC cells. Therefore, we determined whether the anti-proliferative effect of PSA on CRC cells is mediated by the upregulation of IL-8 levels. We analyzed the cell proliferation in the presence of PSA (50 or 100 μg/ml PSA) and anti-human IL-8 neutralizing antibody (10 μg/ml PSA) for 48 h. The result showed that the blocking of IL-8 could abolish the anti-proliferative effect of PSA on CRC as shown in **Figure 2C**. These results suggested that *B. fragilis* PSA may suppress cell proliferation by the induction of IL-8 production from CRC cells.

### PSA Markedly Impairs the Migration and Invasion of CRC Cells by Suppression of the EMT Process

**FIGURE 3:**
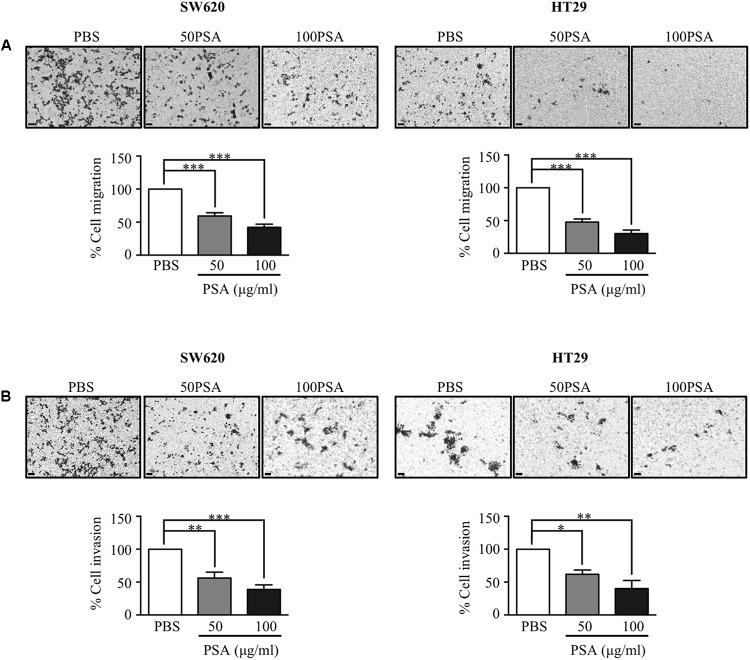
Polysaccharide A suppresses the migration and invasion of colorectal cancer cells. Colorectal cancer cell migration was observed by a Transwell migration assay. The cells were seeded in a Transwell and incubated for 24 h in serum-free media containing 50 or 100 μg/ml PSA. The migratory cells were stained by crystal violet on the surface of Transwell inserts, and the stained cell number was quantified by ImageJ software. The image was obtained by an inverted microscope (magnification: 100×) **(A)**. For invasion assay of CRC cell lines, the experimental process was the same as that described for the Transwell migration assay but in this case the insert of the Transwell was pre-coated with Matrigel for 1 h prior to the assay. The invasive cells were stained on the surface of the Transwell insert, and the stained cell number was quantified by ImageJ software. The image was obtained by an inverted microscope (magnification: 100×) **(B)**. The images are representative of three independent experiments. Data represent the mean ± SD from three independent experiments (^∗^*P* < 0.05, ^∗∗^*P* < 0.005, ^∗∗∗^*P* < 0.0005).

Metastasis to secondary organs is the main cause of cancer-related mortality in CRC patients and it is initiated by migration and invasion of cancer cells (Vatandoust et al., 2015). To evaluate the effect of PSA on CRC cell migration, we performed a Transwell migration assay. The cells were grown in a Transwell insert with serum-free media containing either PBS control or PSA (10, 50, or 100 μg/ml) for 24 h. The results showed that HT29 cell migration was slightly suppressed by 10 μg/ml PSA treatment (**Supplementary Figure S2A**). The percentage of migratory cells was significantly reduced following 50 or 100 μg/ml PSA treatment in a dose-dependent manner for both cell lines (**Figure 3A**). Furthermore, to determine the effect of PSA on CRC cell invasion, Matrigel-coated Transwell assays were performed. As shown in **Figure 3B**, the percentage of invasive cells (both SW620 and HT29) was reduced following 50 or 100 μg/ml PSA treatment in a dose-dependent manner, while 10 μg/ml PSA could slightly suppress the invasion of HT29 cell (**Supplementary Figure S2B**). Collectively, these data demonstrated that PSA may impair the migration and invasion of CRC cells.

**FIGURE 4:**
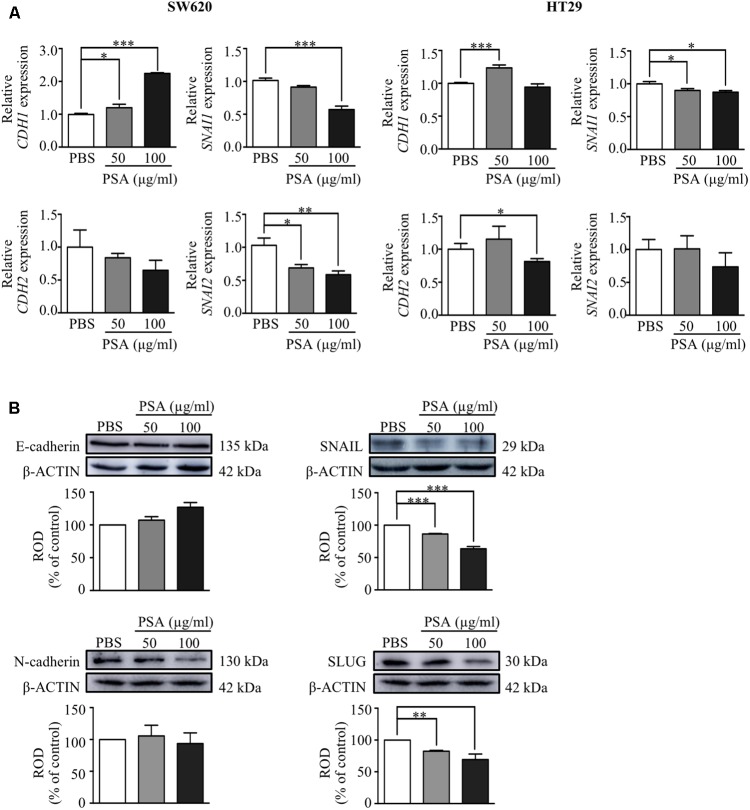
Polysaccharide A upregulates the expression of epithelial cell markers and downregulates the expression of mesenchymal cell markers of colorectal cancer cells. Relative mRNA expression levels of epithelial cell marker; *CDH1* and mesenchymal cell markers; *CDH2*, *SNAI1*, and *SNAI2* were measured in SW620 and HT29 cells after treatment with 50 or 100 μg/ml PSA for 24 h **(A)**. Protein levels of epithelial (*E*-cadherin; encoded by *CDH1*) and mesenchymal (*N*-cadherin; encoded by *CDH2*, SNAIL; encoded by *SNAI1*, and SLUG; encoded by *SNAI2*) cell markers were detected in SW620 cells by western blot analysis; β-actin was used as the loading control and was shared for both N-cadherin and SLUG in the same blot **(B)**. The values are expressed as the mean ± SD of three independent experiments (^∗^*P* < 0.05, ^∗∗^*P* < 0.005, ^∗∗∗^*P* < 0.0005). ROD, relative optical density.

We further determined whether the inhibition of migration and invasion of CRC cells by PSA treatment was related with the EMT process by examining the effect on the gene and protein expression level of the epithelial cell marker; *E*-cadherin (encoded by *CDH1*) and mesenchymal cell markers; *N*-cadherin (encoded by *CDH2*), snail family transcriptional repressor 1 or SNAIL (encoded by *SNAI1*), and snail family transcriptional repressor 2 or SLUG (encoded by *SNAI2*) using RT-PCR and western blot analysis, respectively. The results showed that *CDH1* expression was upregulated while *SNAI1* expression was downregulated by PSA in both cell lines. In addition, the expression level of *CDH2* was significantly decreased by treatment with 100 μg/ml PSA in HT29 cells, and the *SNAI2* expression level was downregulated in a dose-dependent manner by PSA treatment in SW620 cells (**Figure 4A**). Consistently, the protein levels of the mesenchymal markers SNAIL and SLUG were dramatically reduced by PSA treatment in SW620 cells. However, the protein levels of *E*-cadherin and *N*-cadherin did not show a significant difference compared with those in the control (**Figure 4B**). These results suggested that the inhibition of CRC cell migration and invasion by PSA may be mediated by suppression of the EMT process.

### Effects of PSA on SW620 Cells Are Mediated by TLR2 Signaling

**FIGURE 5:**
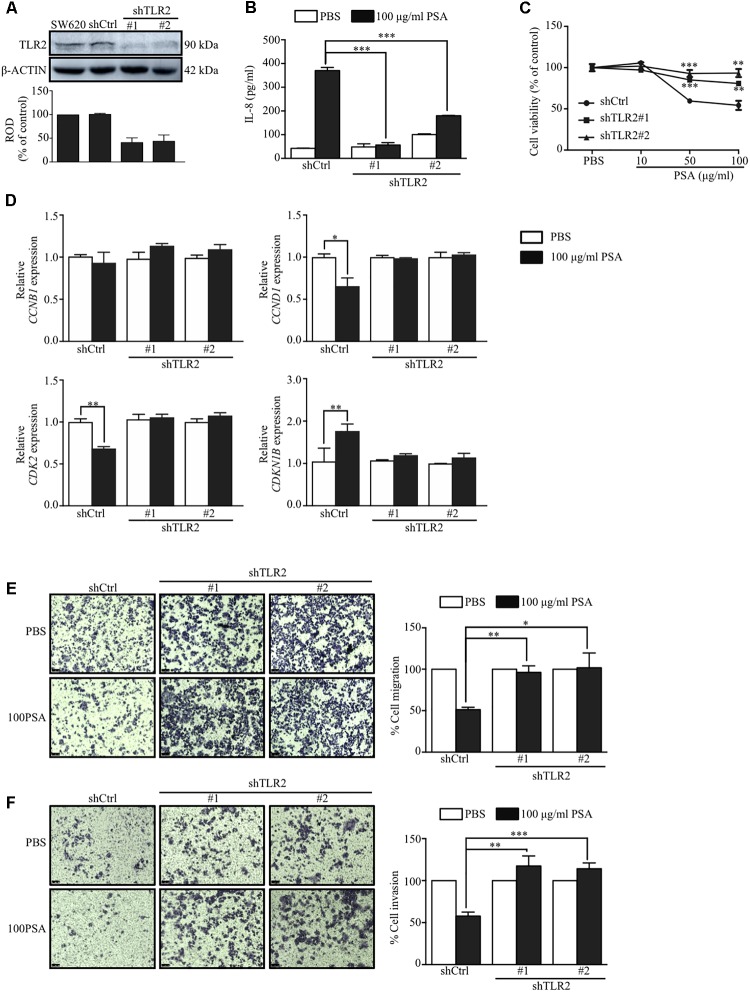
TLR2 signaling mediates the effects of PSA on activation of IL-8 production, and the suppression of proliferation, migration, and invasion of SW620 cells. SW620 colorectal cancer cells were transduced with either nonsense control (shCtrl) or two different TLR2 shRNAs targeting different regions of TLR2 (shTLR2#1 or shTLR2#2), and the transfected cells were selected with puromycin. The cell lysate was obtained from the transduced cells and western blot analysis was performed to analyze the TLR2 protein level; ROD, relative optical density, β-actin was used as the loading control **(A)**. The shCtrl or two different clones of shTLR2-transduced cells were treated with 100 μg/ml PSA for 24 h. The production of IL-8 was assessed by ELISA **(B)**. The shCtrl and shTLR2-transduced cells were treated with 10, 50, or 100 μg/ml PSA for 48 h to determine the cell viability by the MTT assay **(C)**. The shCtrl and shTLR2-transduced cells were treated with 100 μg/ml PSA for 24 h. Relative mRNA expression levels of cell cycle-related genes (*CCNB1*, *CCND1*, *CDK2*, and *CDKN1B*) were measured **(D)**. Cell migration was observed by a Transwell migration assay **(E)**, cell invasion was also evaluated by a Matrigel-coated Transwell assay **(F)**. Data are shown as the mean ± SD from three independent experiments (^∗^*P* < 0.05, ^∗∗^*P* < 0.005, ^∗∗∗^*P* < 0.0005).

The induction of cytokine production in immune cells by *B. fragilis* PSA is TLR2-dependent (Round et al., 2011; Dasgupta et al., 2014). Our data showed that the PSA induced the production of IL-8 and suppressed the proliferation, migration, and invasion of SW620 cells more than that of HT29 cells. We first compared the expression levels of TLR2 in both cell lines and observed that protein levels of TLR2 in SW620 and HT29 were significantly different. The SW620 showed higher TLR2 expression than HT29 (**Supplementary Figure S3**). The high expression of TLR2 may lead to the higher IL-8 production in SW620 than HT29 in response to *B. fragilis* PSA (**Figure 1B**). We next determined whether the induction of IL-8 production by PSA is mediated by TLR2 signaling using SW620 as a model cell line. To determine the requirement of TLR2 for PSA effect on SW620 cells, we used two different TLR2 shRNAs targeting different regions of TLR2 to inhibit the expression of *TLR2* in SW620. The suppression of TLR2 was determined from the transduced cell lysates by western blot using anti-TLR2 antibody, which demonstrated that the TLR2 protein level was decreased by more than 50% in TLR2 shRNA-transduced (shTLR2) cells compared with the nonsense control-transduced (shCtrl) cells (**Figure 5A**). We found that PSA could not activate the production of IL-8 in shTLR2 cells to the levels detected in shCtrl cells (**Figure 5B**). These data suggested that TLR2 is indeed required for *B. fragilis* PSA to activate IL-8 production by CRC cells.

We further explored whether the suppressive effects of PSA on the proliferation, migration, and invasion of CRC cells are also mediated by TLR2. The effect of PSA on these processes were compared between shTLR2 cells and shCtrl cells. The result showed that treatment with 50 or 100 μg/ml PSA did not reduce the viability of shTLR2 cells relative to that of shCtrl cells (**Figure 5C**). In addition, we compared the expressions of cell cycle-related genes between the shCtrl cells and shTLR2 cells under 100 μg/ml PSA treatment. The result showed that the expression of *CCND1* and *CDK2* was downregulated in shCtrl cells, but was rescued in shTLR2 cells by PSA treatment. The expression of *CDKN1B* was upregulated in shCtrl cells, however it was not changed in shTLR2 cells by PSA treatment (**Figure 5D**). Furthermore, PSA could not suppress the migration (**Figure 5E**) and invasion (**Figure 5F**) of shTLR2 cells as it did in shCtrl cells. These findings demonstrated that the effects of *B. fragilis* PSA on the proliferation, migration, and invasion of CRC cells are mediated by TLR2 signaling.

## Discussion

It is now well-established that *B. fragilis* can modulate the production of immune cells, particularly dendritic cells and T cells. Colonization of *B. fragilis*, a human commensal bacterium, has been shown to promote immune development and correct the balance between Th1 and Th2 cells in a germ-free mouse model via its zwitterionic capsular molecule PSA (Mazmanian et al., 2005). The ability of PSA to direct immune system regulation may be explained by its ability to activate the production of cytokines. Treatment with purified PSA from *B. fragilis* has been shown to stimulate the production of IFN-γ, IL-12 (Mazmanian et al., 2005), IL-10, and TGF-β (Mazmanian et al., 2005, 2008; Ochoa-Reparaz et al., 2010a) in co-culture of mouse dendritic cells and T cells. Moreover, *B fragilis* PSA directly interacts with plasmacytoid dendritic cells or T cells to promote IL-10 production, thereby limiting pathogenic inflammation in gut and distant tissue as brain (Mazmanian et al., 2008; Ochoa-Reparaz et al., 2010b; Dasgupta et al., 2014). In addition, purified PSA could directly induce an inflammatory response by activating the production of tumor necrosis factor-alpha (TNF-α) and IL-12 from RAW macrophages and bone marrow-derived dendritic cells (BMDCs) via TLR2-dependent signaling (Wang et al., 2006). However, PSA was also shown to reduce the TNF-α level in a co-culture of infected BMDCs and T cells with live *Helicobacter hepaticus* (Mazmanian et al., 2008). Furthermore, PSA could activate the production of IL-8 by TLR2-transfected human embryonic kidney cells (Wang et al., 2006).

Intestinal inflammation can increase the risk of CRC development by promoting an imbalanced immune state (Danese et al., 2011). Although *B. fragilis* PSA can reduce the intestinal inflammation to potentially suppress CRC progression, the effect of *B. fragilis* PSA on CRC cells has not been elucidated. CRC cells are known to produce several pro-inflammatory cytokines, including IL-8, in response to the bacterial lipopolysaccharide, lipoteichoic acid, and peptidoglycan (Schuerer-Maly et al., 1994). However, the effect of *B. fragilis* PSA on the cytokine production of CRC cells has thus far remained unknown. In this study, we provide the first demonstration that PSA could activate the production of IL-8 from CRC cells (SW620 and HT29). IL-8 (encoded by *CXCL8*) is a member of the CXC chemokine family, which functions as a neutrophil chemoattractant (Baggiolini et al., 1989) and shows angiogenic activities (Koch et al., 1992). A previous study showed that IL-8 acts in an autocrine or paracrine manner to inhibit non-small cell lung cancer proliferation (Wang et al., 1996). Interestingly, our group found that *B. fragilis* could prevent colon cancer development in an AOM/DSS-induced mouse model (Lee, 2018, unpublished data). This observation led us to hypothesize that PSA from *B. fragilis* might inhibit CRC cell proliferation. In this study, we found that PSA has an anti-proliferative effect on CRC cells and this effect is mediated by the IL-8 production by CRC cells. Beside the anti-proliferative effect, PSA also suppresses the migration, and/or invasion of CRC cells. In this study, we confirmed that PSA has a protective role against CRC progression by suppressing cell proliferation, migration, and invasion.

Uncontrolled cell proliferation is a hallmark of cancer cells (Hanahan and Weinberg, 2011), which occurs by avoiding checkpoint pathways in the cell cycle (Bresciani, 1968). To undergo complete cell division, the cell has to process through four stages of the cell cycle—G1, S, G2, and M phase—which are normally regulated by checkpoint controls (Hartwell and Kastan, 1994). Each checkpoint is regulated by different cell cycle regulatory proteins such as cyclins and cyclin-dependent kinases, which form a complex in regulating the cell cycle. These cell cycle proteins have been shown to be promising targets in cancer therapy (Otto and Sicinski, 2017). We here demonstrated that the proliferation of CRC cells was inhibited through the suppression of cell cycle genes by PSA treatment.

Moreover, migration and invasion are the initial steps in the metastasis of cancer cells. The transition of epithelial cancer cells to mesenchymal cells, or EMT, initiates the migration of cancer cells to secondary sites, in a process known as metastasis (van Zijl et al., 2011). Metastasis to secondary organ such as liver and lung are the main cause of cancer-related mortality in CRC patients (Vatandoust et al., 2015). Thus, EMT is strongly associated with the migration and invasion of cancer cells (Kalluri and Weinberg, 2009; Thiery et al., 2009). During the EMT, the expression levels of epithelial proteins, *E*-cadherin (encoded by *CDH1*) as a hallmark of EMT, and other cell junction proteins such as claudins and occludin are downregulated. Meanwhile, mesenchymal proteins such as *N*-cadherin (encoded by *CDH2*) are upregulated to promote the movement of cells. The repression of epithelial gene expression and the activation of mesenchymal gene expression are modulated by three master regulators, including SNAIL, which directly binds and controls the expression of *CDH1* gene, TWIST, and zinc-finger E-box-binding (ZEB) transcription factors (Lamouille et al., 2014). Since we found that PSA could suppress CRC cell migration and invasion, we further determined whether this effect was related with EMT. Indeed, we found that the migration and invasion of CRC cells were reduced by the inhibition of the EMT process, particularly via regulation of the transcription factor SNAIL.

The effect of *B. fragilis* PSA on dendritic cells and T cells is largely mediated by TLR2 (Round et al., 2011; Dasgupta et al., 2014), which has been implicated in the function of *B*. *fragilis* PSA and regulation of immune homeostasis (Wang et al., 2006). TLR2 is one of the bacterial molecule recognition receptors (Janssens and Beyaert, 2003). Previous studies have shown that *B. fragilis* PSA could induce the production of cytokines by immune cells (Round et al., 2011; Dasgupta et al., 2014) and IL-8 by human embryonic kidney cells in a TLR2-signaling dependent manner (Wang et al., 2006). Although TLR signaling has been correlated with CRC, its specific role has been controversial (Li et al., 2014). One study showed that TLR2 did not affect the susceptibility to developing CRC in mice (Salcedo et al., 2010), whereas another study showed that TLR2 signaling protects against the development of tumor formation in a mouse model of colitis-induced cancer (Lowe et al., 2010). Therefore, to explore the requirement of TLR2 for the protective effect of PSA effect on CRC cells, we transduced an shRNA targeting *TLR2* (shTLR2) into the SW60 cell line to deplete the expression of TLR2 protein in CRC cell. The results showed that *B*. *fragilis* PSA no longer induced the production of IL-8 and could not suppress cell proliferation, migration, or invasion of CRC cells in a *TLR2*-deficient condition. These data suggest that the inhibitory effects of PSA on CRC cell growth, migration, and invasion are indeed TLR2-dependent. However, whether PSA directly binds to TLR2 and the precise molecular mechanism driving the action of PSA on CRC cells remains to be addressed in more detailed studies.

Colorectal cancer is one of the primary contributors of cancer mortality worldwide, and metastasis to a secondary tissue is one of crucial events associated with the high mortality rate (Enquist et al., 2014; Siegel et al., 2016). Therefore, *B. fragilis* PSA may be a potential candidate to target the inhibition of CRC cell migration and invasion, which are the initial steps of cancer cell metastasis. However, confirmation of the present results in other CRC cell lines and in an *in vivo* model should be performed to validate the effect of *B. fragilis* PSA on CRC progression. Moreover, the presence of *B. fragilis* in patients with CRC should be elucidated to determine the correlation between microbiota and cancer progression. Nevertheless, our findings reveal that *B. fragilis* PSA might be considered as a potential molecule for CRC treatment.

## Author Contributions

PS designed and performed the experiments and analyzed the data. PS and YKL wrote the manuscript. SL, KC, and INS assisted in performing some experiments. HYK contributed essential ideas and discussion. HYK and YKL supervised the work.

## Conflict of Interest Statement

The authors declare that the research was conducted in the absence of any commercial or financial relationships that could be construed as a potential conflict of interest.

## Supplementary Material

FIGURE S1 Polysaccharide A inhibits the proliferation of patient-derived primary-like CRC cells. Patient-derived primary-like CRC cells were treated with PSA at 50 or 100 μg/ml. MTT solution was added to the cell culture to detect cell viability at 72 h of treatment. The cell viability (percentage) was compared with that of the PBS-treated group (control). The data are shown as the mean ± SD from two independent experiments (^∗∗^*P* < 0.005).

FIGURE S2 Low concentration of PSA slightly suppresses the migration and invasion of CRC cells. Migration of CRC cells was observed by a Transwell migration assay. The cells were seeded in a Transwell and incubated for 24 h in serum-free media containing 10 μg/ml PSA. The migratory cells were stained by crystal violet on the surface of the insert of the Transwell, and the stained cell number was quantified by ImageJ software. The image was obtained by an inverted microscope (magnification: 100×) **(A)**. For invasion assay, the insert of the Transwell was pre-coated with Matrigel for 1 h prior to the assay. The invasive cells were stained on the surface of the Transwell insert, and the stained cell number was quantified by ImageJ software. The image was obtained by an inverted microscope (magnification: 100×) **(B)**. The images are representative of three independent experiments. Data represent the mean ± SD from three independent experiments (^∗^*P* < 0.05).

FIGURE S3 SW620 cells show higher expression of TLR2 than HT29 cells. Protein levels of TLR2 was detected in SW620 and HT29 cells by western blot analysis; β-actin was used as the loading control. ROD, relative optical density.
