# Supplementary figures and images for: Toll-Like Receptor 2-Mediated Suppression of Colorectal Cancer Pathogenesis by Polysaccharide A From Bacteroides fragilis
Source: Front Microbiol. 2018 Jul 17;9:1588. doi: 10.3389/fmicb.2018.01588 (PMC6056687; doi:10.3389/fmicb.2018.01588)

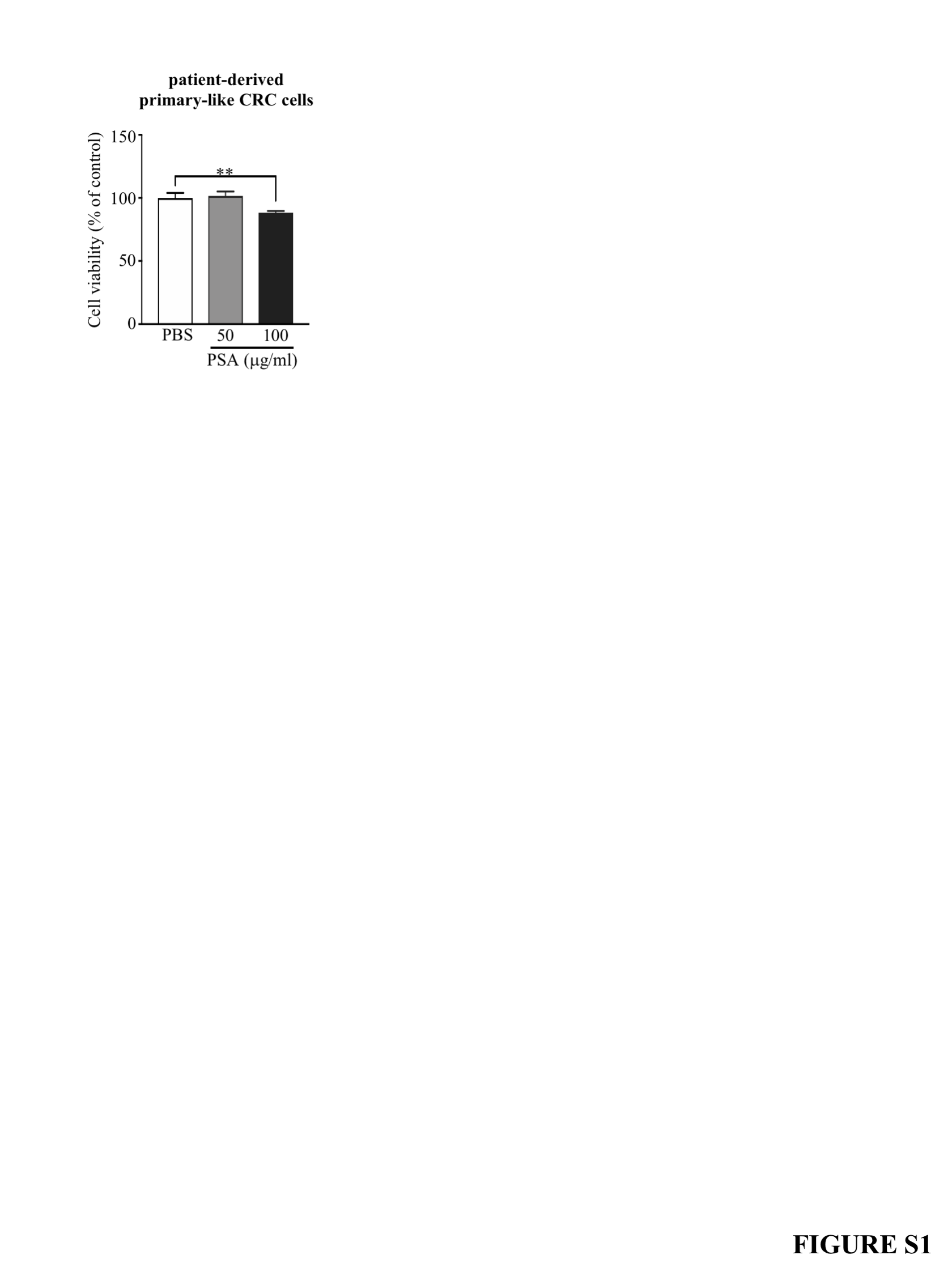

Supplement: FIGURE S1 — Polysaccharide A inhibits the proliferation of patient-derived primary-like CRC cells. Patient-derived primary-like CRC cells were treated with PSA at 50 or 100 μg/ml. MTT solution was added to the cell culture to detect cell viability at 72 h of treatment. The cell viability (percentage) was compared with that of the PBS-treated group (control). The data are shown as the mean ± SD from two independent experiments (∗∗P < 0.005). [file Image_1.tiff]

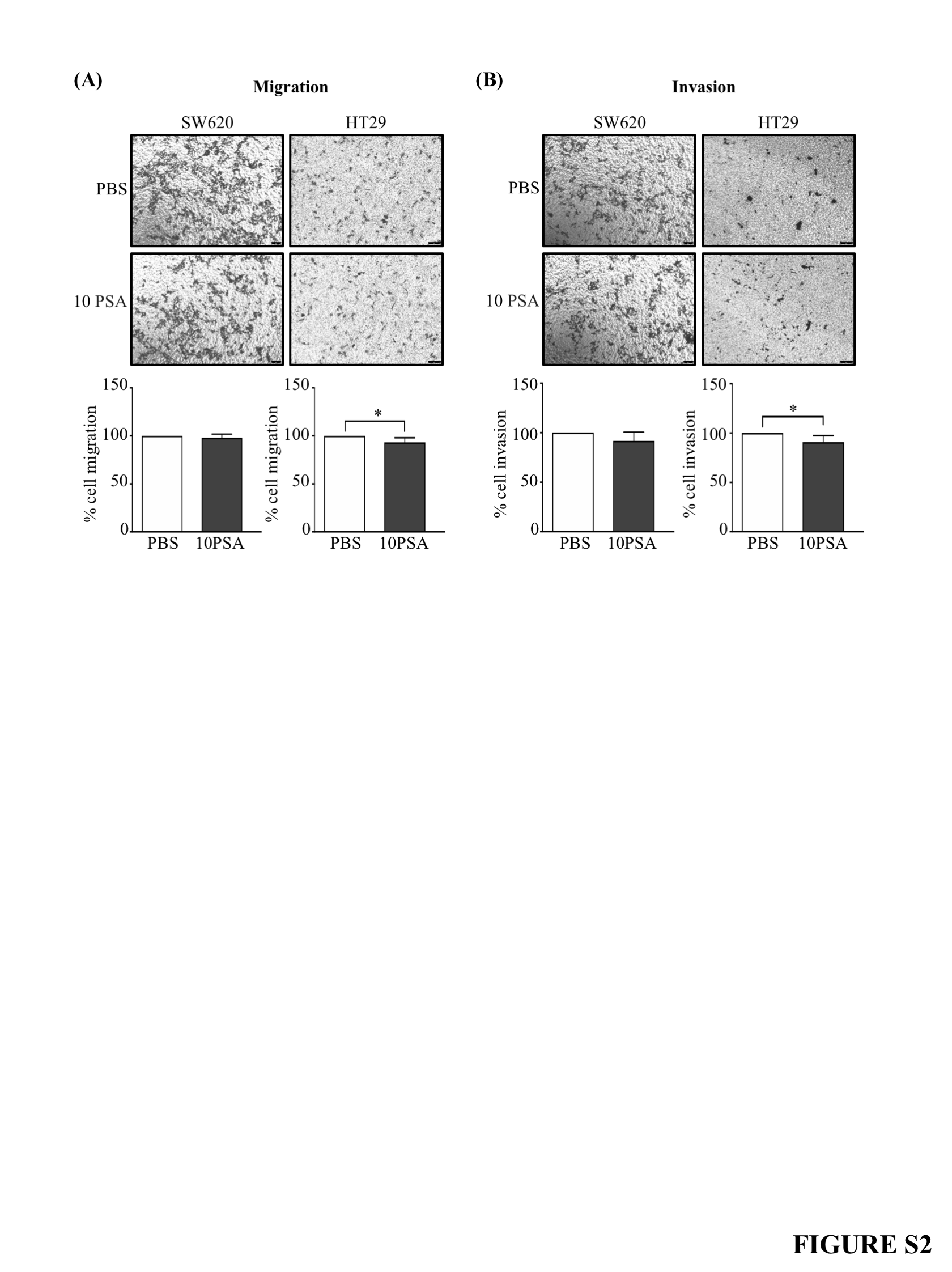

Supplement: FIGURE S2 — Low concentration of PSA slightly suppresses the migration and invasion of CRC cells. Migration of CRC cells was observed by a Transwell migration assay. The cells were seeded in a Transwell and incubated for 24 h in serum-free media containing 10 μg/ml PSA. The migratory cells were stained by crystal violet on the surface of the insert of the Transwell, and the stained cell number was quantified by ImageJ software. The image was obtained by an inverted microscope (magnification: 100×) (A). For invasion assay, the insert of the Transwell was pre-coated with Matrigel for 1 h prior to the assay. The invasive cells were stained on the surface of the Transwell insert, and the stained cell number was quantified by ImageJ software. The image was obtained by an inverted microscope (magnification: 100×) (B). The images are representative of three independent experiments. Data represent the mean ± SD from three independent experiments (∗P < 0.05). [file Image_2.tiff]

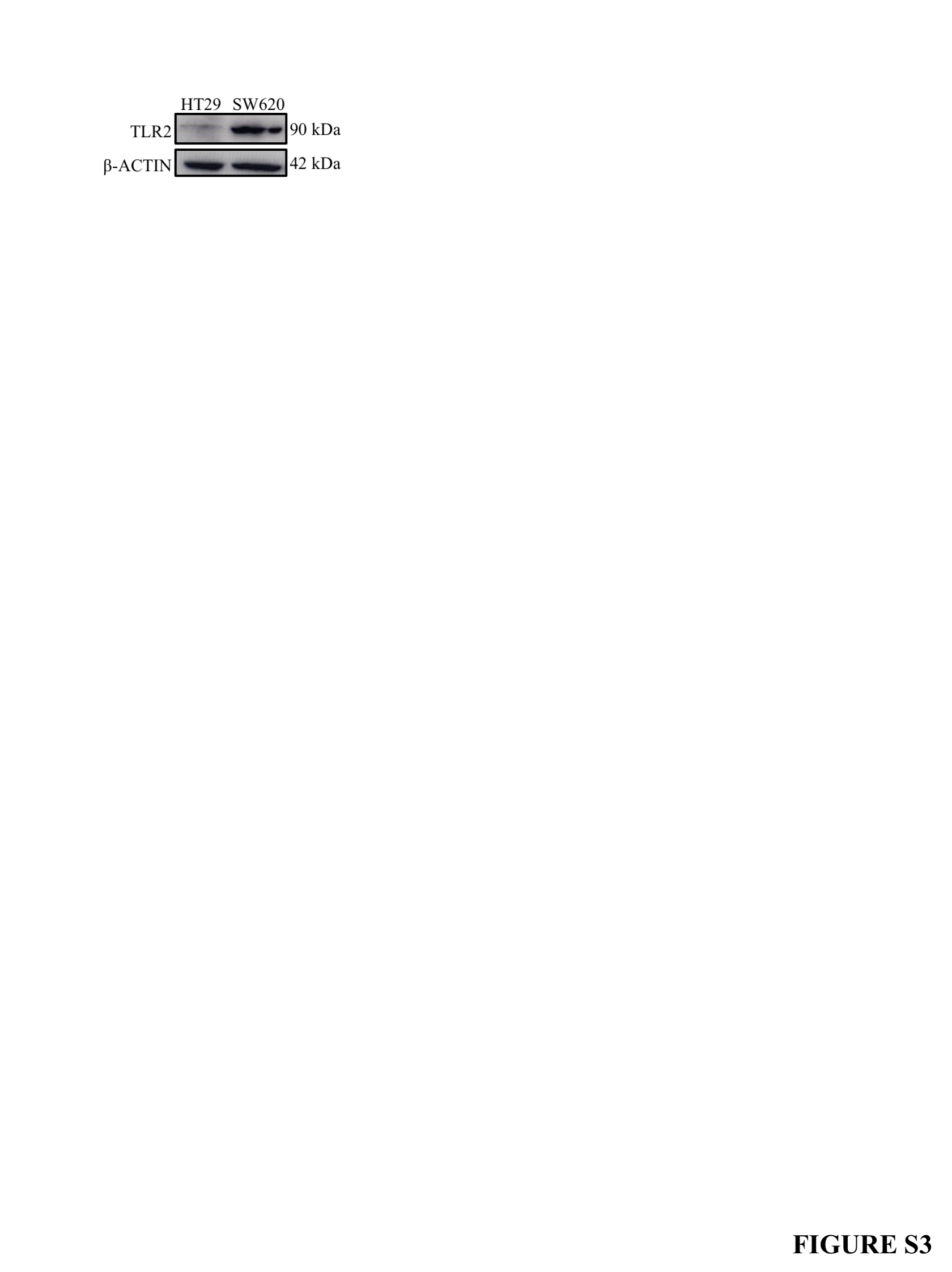

Supplement: FIGURE S3 — SW620 cells show higher expression of TLR2 than HT29 cells. Protein levels of TLR2 was detected in SW620 and HT29 cells by western blot analysis; β-actin was used as the loading control. ROD, relative optical density. [file Image_3.tiff]
